# Establishment and external verification of an oxidative stress-related gene signature to predict clinical outcomes and therapeutic responses of colorectal cancer

**DOI:** 10.3389/fphar.2022.991881

**Published:** 2023-02-13

**Authors:** Sha Cao, Cheng Chen, Dezhi Gu, Zhengdong Wang, Guanghui Xu

**Affiliations:** ^1^ Department of Oncology, The First People’s Hospital of Lianyungang, Lianyungang, China; ^2^ Department of Gastrointestinal Surgery, The First People’s Hospital of Lianyungang, Lianyungang, China

**Keywords:** colorectal cancer, oxidative stress, prognosis, antitumor immunity, drug sensitivity

## Abstract

**Objective:** Accumulated evidence highlights the biological significance of oxidative stress in tumorigenicity and progression of colorectal cancer (CRC). Our study aimed to establish a reliable oxidative stress-related signature to predict patients’ clinical outcomes and therapeutic responses.

**Methods:** Transcriptome profiles and clinical features of CRC patients were retrospectively analyzed from public datasets. LASSO analysis was used to construct an oxidative stress-related signature to predict overall survival, disease-free survival, disease-specific survival, and progression-free survival. Additionally, antitumor immunity, drug sensitivity, signaling pathways, and molecular subtypes were analyzed between different risk subsets through TIP, CIBERSORT, oncoPredict, etc. approaches. The genes in the signature were experimentally verified in the human colorectal mucosal cell line (FHC) along with CRC cell lines (SW-480 and HCT-116) through RT-qPCR or Western blot.

**Results:** An oxidative stress-related signature was established, composed of *ACOX1*, *CPT2*, *NAT2*, *NRG1*, *PPARGC1A*, *CDKN2A*, *CRYAB*, *NGFR*, and *UCN*. The signature displayed an excellent capacity for survival prediction and was linked to worse clinicopathological features. Moreover, the signature correlated with antitumor immunity, drug sensitivity, and CRC-related pathways. Among molecular subtypes, the CSC subtype had the highest risk score. Experiments demonstrated that *CDKN2A* and *UCN* were up-regulated and *ACOX1*, *CPT2*, *NAT2*, *NRG1*, *PPARGC1A*, *CRYAB*, and *NGFR* were down-regulated in CRC than normal cells. In H_2_O_2_-induced CRC cells, their expression was notably altered.

**Conclusion:** Altogether, our findings constructed an oxidative stress-related signature that can predict survival outcomes and therapeutic response in CRC patients, thus potentially assisting prognosis prediction and adjuvant therapy decisions.

## Introduction

Modulation of redox homeostasis is essential for maintaining normal cellular function and ensuring cell survival. Tumor cells are characterized by high levels of oxidative stress that is a state of imbalance between oxidation and antioxidation (Donohoe et al., 2019). Accumulated evidence suggests that oxidative stress exhibits dual roles in tumor progression (Yang and Chen, 2021). Reactive oxygen species (ROS) exhibits antitumor effects by heightening tumor cell apoptosis, necrosis, and ferroptosis and strengthening the immune surveillance capacity of immune cells (Gorrini et al., 2013). Instead, ROS promotes tumor progression *via* triggering DNA damage and genomic changes, activating proliferation- and epithelial–mesenchymal transition-related pathways, and remodeling the tumor microenvironment for tumor invasion and metastases (Falone et al., 2019).

Colorectal cancer (CRC) remains the third most diagnosed cancer (10.0%), and the second leading cause of cancer death (9.4%) worldwide, according to GLOBOCAN 2020 estimates (Sung et al., 2021). Approximately 50% of patients die from tumor metastases (Li et al., 2022). Currently, systemic treatment options comprise adjuvant and neoadjuvant chemotherapy, and therapeutic antibodies directed against growth factor receptors (Berlin et al., 2022). Nevertheless, 30–40% of patients relapse despite treatment. A reasonable and effective signature for prognostic assessment of CRC patients is required. Oxidative stress can induce genetic instability and alter cellular processes, leading to CRC (Wei et al., 2021). In a large CRC patient cohort, higher reactive oxygen metabolites exhibit a strong association with more undesirable survival outcomes (Boakye et al., 2020). Cancer cells adapt to chemotherapy-induced oxidative stress using rapidly elevated cellular antioxidant programs, and adaptation of oxidative defense results in therapeutic resistance, a primary barrier to successful cancer treatment (Čipak Gašparović et al., 2021). For instance, SIRT3-mediated SOD2 and PGC-1α trigger chemoresistance in CRC cells (Paku et al., 2021). Moreover, up-regulated NOX-2 and Nrf-2 facilitate 5-fluorouracil resistance of CRC cells (Waghela et al., 2021). Given the crucial roles of oxidative stress in the progression and therapeutic resistance of CRC, this study attempted to construct a reliable oxidative stress-related signature to predict patients’ clinical outcomes and therapeutic responses.

## Materials and methods

### CRC datasets

Transcriptome profiling (RNA-seq) of colon adenocarcinoma (COAD) and rectum adenocarcinoma (READ) was performed, and normal tissue samples were extracted from The Cancer Genome Atlas (TCGA) *via* the Genomic Data Commons (GDC). The raw counts were standardized to count-per-million (CPM) using the edgeR package (Robinson et al., 2010). The threshold was set to 1 to retain genes greater than 1 in 2 or more samples. The copy number variation (CNV) data (masked copy number segment) and somatic mutation data (Varscan2) of CRC samples were downloaded from TCGA. Microarrays of CRC patients in GSE12945 (Staub et al., 2009), GSE39582 (Marisa et al., 2013), and GSE103479 (Allen et al., 2018) were acquired from the Gene Expression Omnibus (GEO). Microarray data were corrected for background and normalized through the robust multichip average (RMA) method. Missing data were imputed through the K-nearest neighbor method.

### Identification of differentially expressed oxidative stress-related genes

Differentially expressed genes between CRC and normal tissues were screened based on the criteria of |log2fold-change|≥1 and adjusted *p* ≤ 0.05 utilizing the edgeR package. Adjusted p was calculated through the Bonferroni and Hochberg method. In total, 1,399 oxidative stress-related genes were extracted from the GeneCards according to relevance score≥7 (Supplementary Table S1). Afterward, differentially expressed oxidative stress-related genes were intersected.

### Prognostic model construction

Univariate cox regression models were established to determine survival-related differentially expressed oxidative stress-related genes with *p* < 0.05. Through the least absolute shrinkage and selection operator method (LASSO), a prognosis gene signature was developed with the glmnet package (Friedman et al., 2010). The risk score was computed by the expression of candidate genes along with their coefficients. TCGA CRC samples were randomly assigned to the training set along with the testing set at 1:1 ratio (Liu et al., 2020). In each set, the median risk score was set as the cut-off value of low- and high-risk subsets.

### Survival analysis

Kaplan–Meier curves along with the log-rank test were conducted on oxidative stress-relevant gene signature and patients’ overall survival (OS), disease-free survival (DFS), disease-specific survival (DSS), and progression-free survival (PFS) based on the clinical data. Uni- and multivariate Cox regression models were established on the gene signature, and clinical parameters and OS with the survival package. Through the survival-ROC package, receiver operator characteristic curves (ROCs) were drawn, followed by the area under the curve (AUC) value.

### Quantification of immune cell infiltration

Immune cell infiltrations were estimated across CRC tissues through Cell Type Identification by Estimating Relative Subsets of RNA Transcripts (CIBERSORT), a deconvolution approach proposed by Newman et al. (2015). The LM22 gene set was set as the reference set. This analysis was repeated 1,000 times, with *p* < 0.05 as the filtering condition.

### Cancer immunity cycle

The cancer immunity cycle containing seven steps reflects the antitumor immunity as previously described (Chen and Mellman, 2013). The enrichment score of these steps was quantified *via* the TIP approach (Xu et al., 2018).

### Analysis of CNV and mutation data

On the basis of the recurrently altered regions derived from the Genomic Identification of Significant Targets in Cancer (GISTIC 2.0) algorithm (Mermel et al., 2011), significant focal regions of gain and loss were identified and scored (G-score). The parameter thresholds were set as gain or loss length>0.1 and *p* < 0.05. Somatic mutation data were analyzed with the maftools package (version 2.6.0) (Mayakonda et al., 2018).

### Drug sensitivity analysis

Drug Sensitivity data were acquired from the Genomics of Drug Sensitivity in Cancer (GDSC) database (www.cancerRxgene.org) (Yang et al., 2013). IC50 values were estimated with the oncoPredict package (Maeser et al., 2021).

### Gene set enrichment analysis

GSEA was carried out through the Java platform (Subramanian et al., 2005). Gene sets of Gene Ontology (GO) and Kyoto Encyclopedia of Genes and Genomes (KEGG) were obtained from the Molecular Signatures Database (Liberzon et al., 2015). Terms with FDR<0.05 after 1,000 permutations were significantly enriched.

### Cell culture and treatment

Human colorectal mucosal cell lines (FHC) and CRC cell lines (SW-480 and HCT-116) were maintained in DMEM with 10% fetal bovine serum, 100 U/ml penicillin, and 100 μg/ml streptomycin in a 37°C humidified incubator with 5% CO_2_. To induce oxidative stress, the cells were administrated H_2_O_2_ in the medium, which was changed daily.

### RT-qPCR

RNA extraction was performed using the TRIzol reagent (Invitrogen, United States) and DNase I, followed by reverse transcription into complementary DNAs (cDNAs) utilizing the Superscript Reverse Transcriptase Kit (Thermo Fisher, United States). RT-qPCR was implemented with the Super SYBR Green Kit (BIO-RAD, United States) using the ABI7300 RT-qPCR system (Applied Biosystems, United States). The primer pairs included *ACOX1*, 5ʹ-TAA​CTT​CCT​CAC​TCG​AAG​CCA-3ʹ (forward), 5ʹ-AGT​TCC​ATG​ACC​CAT​CTC​TGT​C-3ʹ (reverse); *CDKN2A*, 5ʹ-GAT​CCA​GGT​GGG​TAG​AAG​GTC-3ʹ (forward), 5ʹ-CCC​CTG​CAA​ACT​TCG​TCC​T-3ʹ (reverse); *CPT2*, 5ʹ-CAT​ACA​AGC​TAC​ATT​TCG​GGA​CC-3ʹ (forward), 5ʹ-AGC​CCG​GAG​TGT​CTT​CAG​AA-3ʹ (reverse); *CRYAB*, 5ʹ-CCT​GAG​TCC​CTT​CTA​CCT​TCG-3ʹ (forward), 5ʹ-CAC​ATC​TCC​CAA​CAC​CTT​AAC​TT-3ʹ (reverse); *NAT2*, 5ʹ-ACC​TGG​ACC​AAA​TCA​GGA​GAG-3ʹ (forward), 5ʹ-TGT​TCG​AGG​TTC​AAG​CGT​AAA​T-3ʹ (reverse); *NGFR*, 5ʹ-CCT​ACG​GCT​ACT​ACC​AGG​ATG-3ʹ (forward), 5ʹ-CAC​ACG​GTG​TTC​TGC​TTG​T-3ʹ (reverse); *NRG1*, 5ʹ-CGG​TGT​CCA​TGC​CTT​CCA​T-3ʹ (forward), 5ʹ-GTG​TCA​CGA​GAA​GTA​GAG​GTC​T-3ʹ (reverse); *PPARGC1A*, 5ʹ-TCT​GAG​TCT​GTA​TGG​AGT​GAC​AT-3ʹ (forward), 5ʹ-CCA​AGT​CGT​TCA​CAT​CTA​GTT​CA-3ʹ (reverse); *UCN*, 5ʹ-CAA​CCC​TTC​TCT​GTC​CAT​TGA​C-3ʹ (forward), 5ʹ-CGA​GTC​GAA​TAT​GAT​GCG​GTT​C-3ʹ (reverse); and *GAPDH*, 5ʹ-ACA​ACT​TTG​GTA​TCG​TGG​AAG​G-3ʹ (forward), 5ʹ-GCC​ATC​ACG​CCA​CAG​TTT​C-3ʹ (reverse). With GAPDH as an internal control, the relative expression was quantified using the 2^−ΔΔCt^ method.

### Western blot

Protein was extracted from cells using RIPA lysis buffer, and protein concentration was assessed using the Bradford protein assay kit (Keygen, China). Protein samples were subjected to 8 or 12% SDS-PAGE gels and transferred onto PVDF membranes, followed by incubation with the primary antibody of *ACOX1* (1/1,000; ab184032), *CDKN2A* (1/1,000; ab270058), *CPT2* (1/3,000; ab181114), *CRYAB* (1/1,000; ab281561), *NAT2* (1/5,000; ab194114), *NGFR* (1/10,000; ab52987), *NRG1* (1/1,000; ab191139), *PPARGC1A* (1/1,000; ab188102), *UCN* (1/1,000; ab231050), or *GAPDH* (1/1,000; ab125247) at 4°C. The next day, the membrane was incubated with horseradish peroxidase-linked secondary antibodies at room temperature for 1 h. Protein bands were developed using the ECL reagent (Tanon, China), and gray values were quantified *via* ImageJ software.

### Statistical analysis

Statistical analysis was generated through R 3.6.1. Statistical difference between groups was computed with unpaired Student’s t-test, Wilcoxon test, Kruskal–Wallis test, or one-way analysis of variance. Two-tailed *p* < 0.05 was set as statistical difference.

## Results

### Development of an oxidative stress-related gene signature for CRC

**FIGURE 1:**
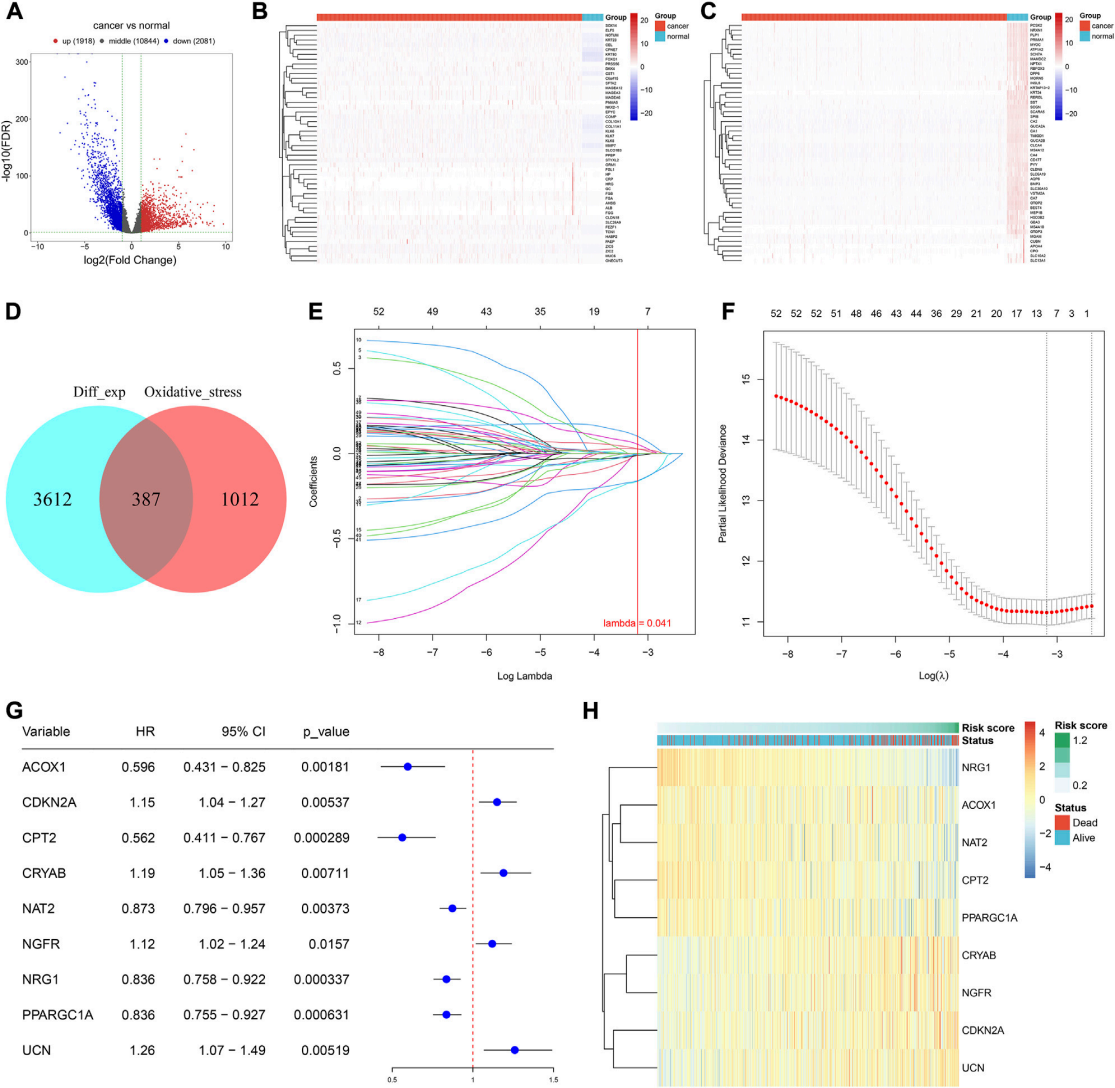
Development of an oxidative stress-related gene signature for CRC. **(A)** Volcano plot of the DEGs between CRC and normal tissues in the TCGA-COAD cohort. Red, up-regulated genes; blue, down-regulated genes. **(B,C)** Heatmaps of up- and down-regulated DEGs in CRC *versus* normal tissues in the TCGA-COAD cohort. **(D)** Venn plot of the DEGs and oxidative stress-related genes. **(E)** Identification of the optimal coefficients of oxidative stress-related genes according to the optimal lambda. X-axis is the log lambda; Y-axis is the coefficient of each variable. **(F)** Optimal partial likelihood deviance along with the optimal lambda. **(G)** Forest plot of the univariate cox-regression results of the oxidative stress-relevant genes within the LASSO model. **(H)** Expression of aforementioned genes among CRC samples. *N* = 597.

In total, there were 1,918 up-regulated genes and 2,081 down-regulated genes in 638 CRC *versus* 51 normal tissues (Figures 1A–C). The detailed information is listed in Supplementary Table S2. From the GeneCards, we extracted 1,399 oxidative stress-related genes. After taking the intersection, 387 differentially expressed oxidative stress-related genes were finally identified (Figure 1D). Among them, 53 genes were significantly correlated with CRC prognosis (Supplementary Table S3). Afterward, candidate genes with regression coefficient≠0 were used for constructing an oxidative stress-related gene signature using the LASSO algorithm (Figures 1E,F). The risk score was computed according to (-0.00277909287793242) * *ACOX1* expression + 0.0280830167034478 * *CDKN2A* expression + (-0.163084055105811) * *CPT2* expression + 0.0548399857226341 * *CRYAB* expression + (-0.0107247779354099) * *NAT2* expression + 0.0267977941327448 * *NGFR* expression + (-0.160185818265943) * *NRG1* expression + (-0.00515077740891848) * *PPARGC1A* expression + 0.10199017424903 * *UCN* expression. For CRC prognosis, *ACOX1*, *CPT2*, *NAT2*, *NRG1*, and *PPARGC1A* were protective factors, and *CDKN2A*, *CRYAB*, *NGFR*, and *UCN* were risk factors (Figure 1G). Figure 1H visualizes the expression of the aforementioned genes across CRC samples.

### The oxidative stress-related gene signature accurately predicts CRC prognosis

**FIGURE 2:**
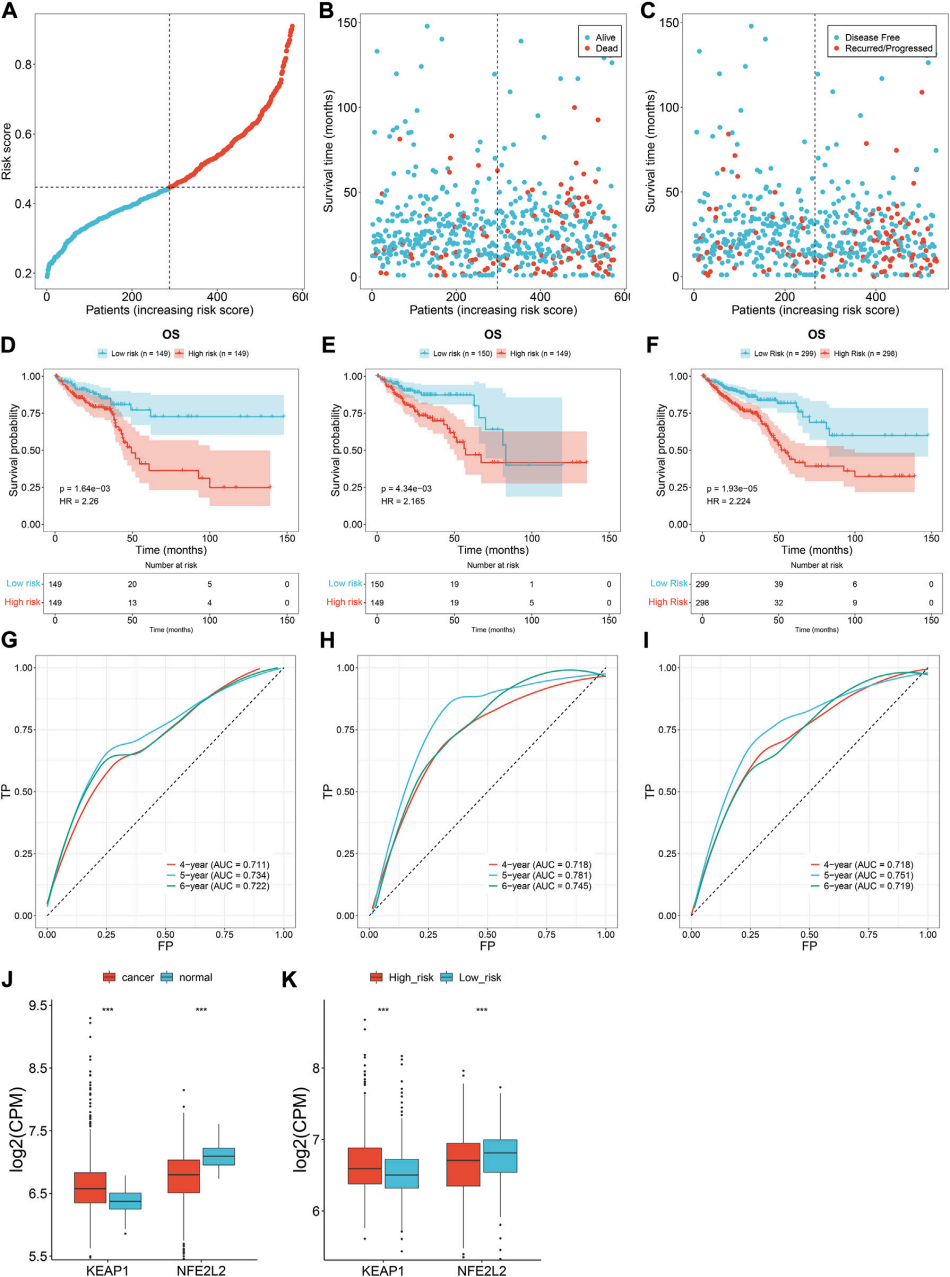
Oxidative stress-related gene signature accurately predicts CRC prognosis. **(A)** Distribution of the risk score derived from the oxidative stress-related gene signature in TCGA CRC patients. The vertical dotted line represents the median value. Red dots, high-risk samples; blue dots, low-risk samples. **(B)** Scatter plots of alive (blue) and dead (red) cases along the increasing risk score. **(C)** Scatter plots of disease-free (blue) and recurred/progressed (red) cases along the increasing risk score. **(D–F)** Kaplan–Meier OS for high- and low-risk subsets within the **(D)** training set, **(E)** the testing set, along with **(F)** the total set. **(G–I)** ROCs under 4-, 5- and 6-year survival for the **(G)** training set, **(H)** the testing set, along with **(I)** the total set. **(J)** Comparison of the expression of two master regulators of oxidative stress (*NRF2* and *KEAP1*) in normal *versus* CRC tissues. **(K)** Comparison of the expression of *NRF2* and *KEAP1* in r high- and low-risk subsets. ***p < 0.001. Total set: *N* = 597; training set: *N* = 298; testing set: *N* = 299.

TCGA patients (*N* = 597) were randomly allocated into the training set (*N* = 298) and testing set (*N* = 299) at 1:1 ratio. Table 1 lists the patients’ clinicopathological characteristics in the total, training along with testing sets. According to the median value, CRC cases were allocated into the high- or low-risk subsets (Figure 2A), with relatively more dead and recurred/progressed cases in the high-risk subset (Figures 2B,C). The OS outcomes of the high-risk subset were significantly decreased in comparison to those of the low-risk subset in the training set (Figure 2D) and the testing set (Figure 2E) along with the total set (Figure 2F). ROCs under 4-, 5-, and 6-year OS of the training set (Figure 2G), the testing set (Figure 2H) along with the total set (Figure 2I) demonstrated the excellent performance of the oxidative stress-related gene signature in predicting CRC prognosis.

**TABLE 1:** Clinical characteristics of CRC patients in the total, training, and testing sets.

Variable	Total set (*N* = 597)	Training set (*N* = 298)	Testing set (*N* = 299)
Age	66.07 ± 12.7	66.52 ± 12.36	65.61 ± 13.03
Status (n, %)			
Alive	472 (79.06)	233 (78.19)	239 (79.93)
Dead	125 (20.94)	65 (21.81)	60 (20.07)
Sex (n, %)			
Male	322 (53.94)	169 (56.71)	153 (51.17)
Female	275 (46.06)	129 (43.29)	146 (48.83)
T stage (n, %)			
T1	20 (3.35)	9 (3.02)	11 (3.68)
T2	103 (17.25)	55 (18.46)	48 (16.05)
T3	408 (68.34)	200 (67.11)	208 (69.57)
T4	64 (10.72)	33 (11.07)	31 (10.37)
Ti	1 (0.17)	1 (0.34)	0 (0)
Unknown	1 (0.17)	0 (0)	1 (0.33)
N stage (n, %)			
N0	337 (56.45)	180 (60.4)	157 (52.51)
N1	147 (24.62)	63 (21.14)	84 (28.09)
N2	110 (18.43)	55 (18.46)	55 (18.39)
Unknown	3 (0.5)	0 (0)	3 (1)
M stage (n, %)			
M0	443 (74.2)	220 (73.83)	223 (74.58)
M1	84 (14.07)	41 (13.76)	43 (14.38)
Unknown	70 (11.73)	37 (12.42)	33 (11.04)
Pathologic stage (n, %)			
Stage I	103 (17.25)	55 (18.46)	48 (16.05)
Stage II	217 (36.35)	117 (39.26)	100 (33.44)
Stage III	175 (29.31)	77 (25.84)	98 (32.78)
Stage IV	87 (14.57)	43 (14.43)	44 (14.72)
Unknown	15 (2.51)	6 (2.01)	9 (3.01)

We also measured the expression of two master regulators of oxidative stress (*NRF2* and *KEAP1*). Compared with normal tissues, up-regulated *KEAP1* and down-regulated *NRF2* were found in CRC tissues at the transcriptional level (Figure 2J), indicating the enhanced oxidative stress during CRC development. Additionally, we observed the difference in *NRF2* and *KEAP1* between high- and low-risk subsets. As shown in Figure 2K, the high-risk subset presented higher *KEAP1* expression and lower *NRF2* expression in comparison to the low-risk subset, demonstrating the heterogeneity in oxidative stress between high- and low-risk CRC patients.

### The oxidative stress-related gene signature correlates to clinical characteristics of CRC

**FIGURE 3:**
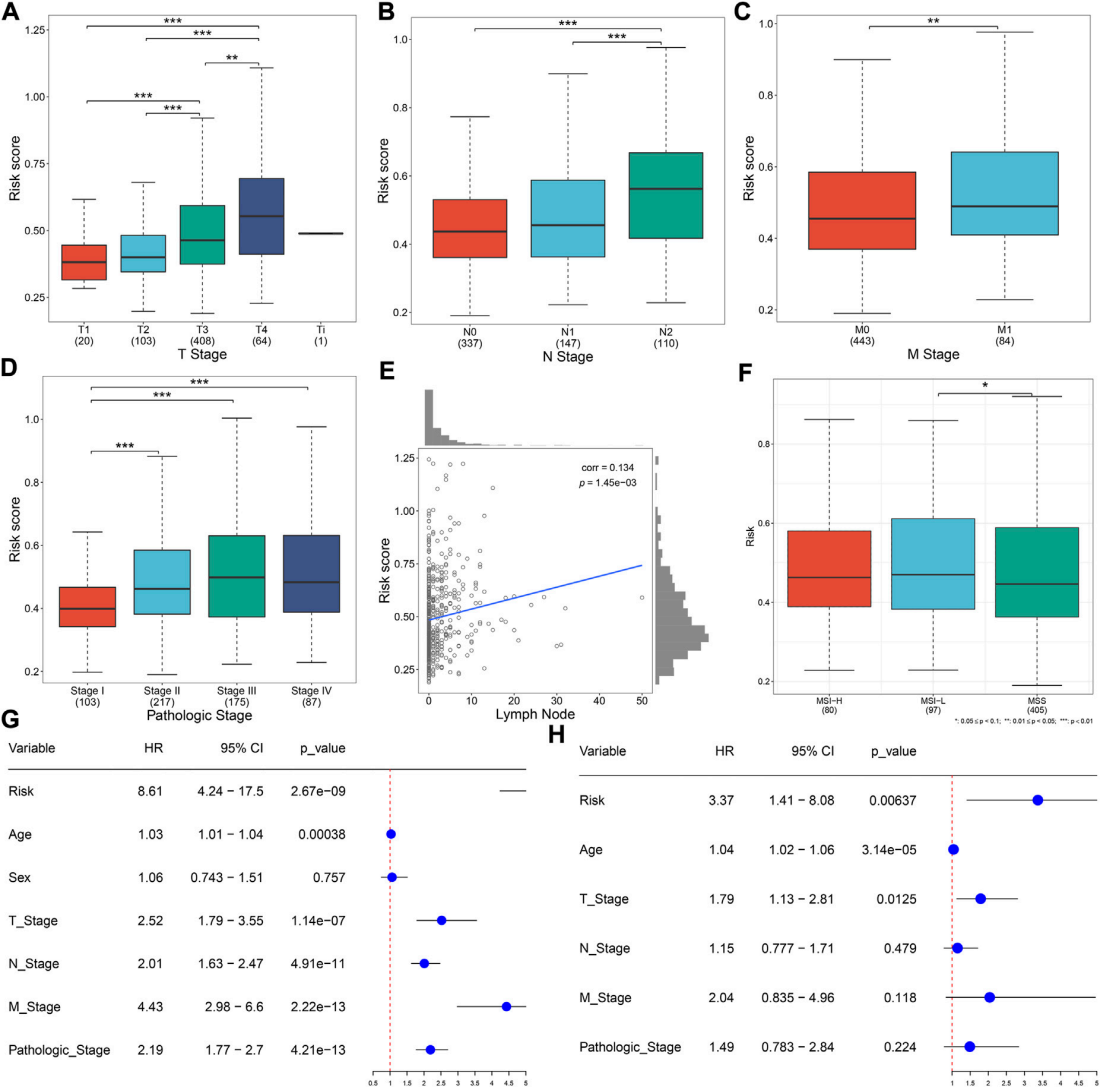
Oxidative stress-related gene signature correlates to clinical characteristics and serves as an independent prognostic factor in TCGA CRC. **(A–D)** Distribution of the risk score derived from the oxidative stress-related gene signature across **(A)** T, **(B)** N, **(C)** M, and **(D)** pathological stage. **(E)** Scatter plot of the correlation between the risk score and lymph node. **(F)** Distribution of the risk score across different microsatellite status. **(G,H)** Forest plots of the uni- and multivariate Cox regression results. *p < 0.05; **p < 0.01; ***p < 0.001. *N* = 597.

Distribution of the risk score derived from the oxidative stress-related gene signature was analyzed across different clinical characteristics. With the increasing TNM and pathological stage, the risk score was dramatically higher (Figures 3A–D). Additionally, the risk score was positively correlated to the lymph node (Figure 3E). Compared with microsatellite-stable (MSS), microsatellite unstable-low (MSI-L) had a significantly higher risk score (Figure 3F). Overall, the oxidative stress-related gene signature was correlated to a more serious pathological status.

### The oxidative stress-related gene signature acts as an independent prognostic factor of CRC patients

**FIGURE 4:**
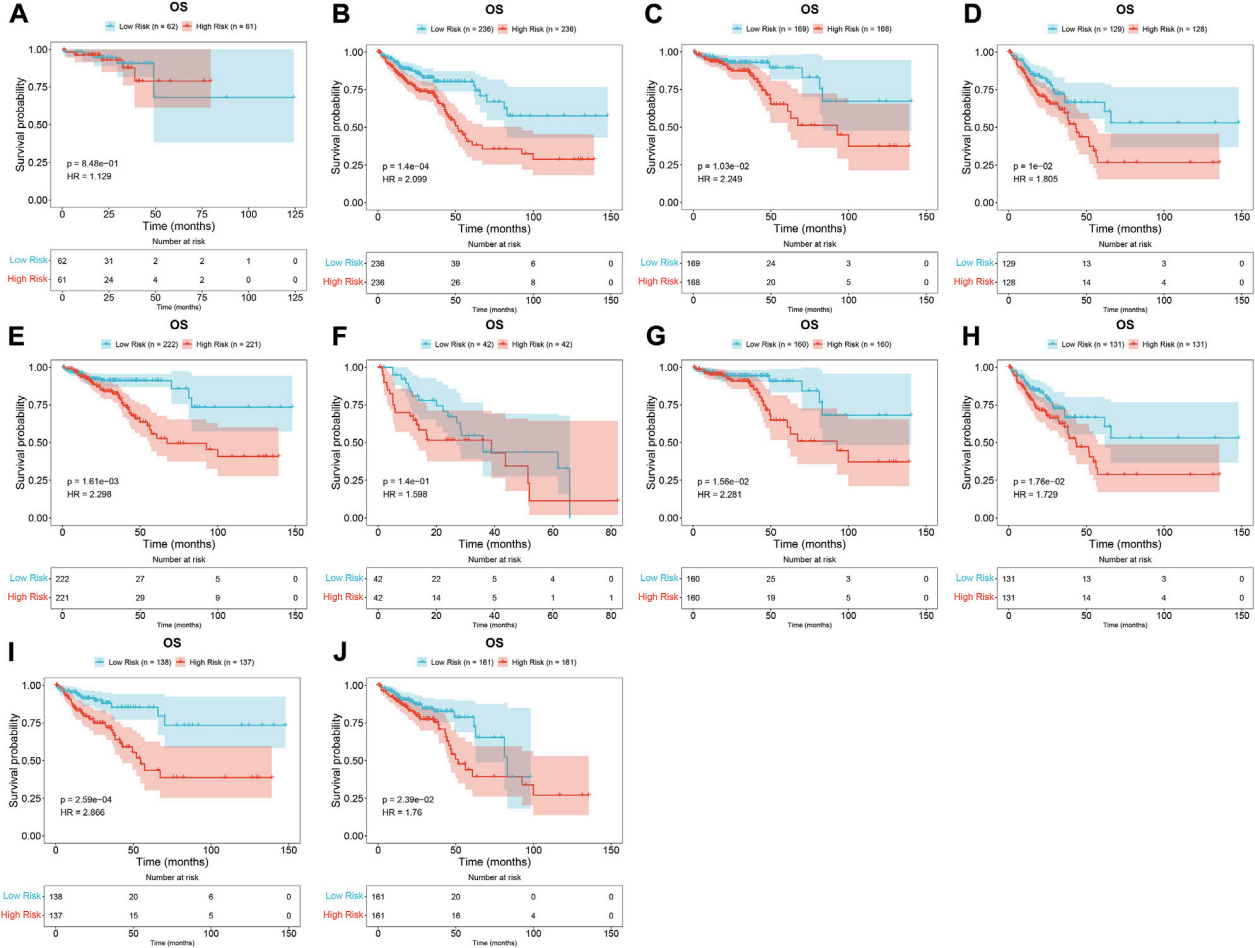
Oxidative stress-related gene signature is independent of other clinical features in predicting CRC patients’ prognosis. **(A–J)** Subgroup analysis for the OS difference between high- and low-risk subsets in each subgroup stratified by **(A,B)** T (T1–2 and T3–4), **(C,D)** N (N0 and N1–2), **(E,F)** M (M0 and M1), **(G,H)** pathological stage (stage I–II, stage III–IV), or **(I,J)** sex (female and male) in the TCGA dataset. *N* = 597.

Uni- and multivariate Cox regression models demonstrated that the risk score acted as an independent risk factor of CRC survival (Figures 3G,H). CRC patients were stratified by the T stage (T1–2 and T3–4), N stage (N0 and N1–2), M stage (M0 and M1), pathological stage (stage I–II and stage III–IV), or sex (female and male). In each subgroup, OS (Figures 4A–J), DFS (Supplementary Figures 1A–J), DSS (Supplementary Figure S2A–J), and PFS (Supplementary Figure S3A–J) of the high-risk subset were dramatically decreased in comparison with those of the low-risk subset. Hence, this oxidative stress-relevant gene signature was independent of other clinical characteristics in predicting CRC patients’ prognosis.

### External verification of the oxidative stress-related gene signature

**FIGURE 5:**
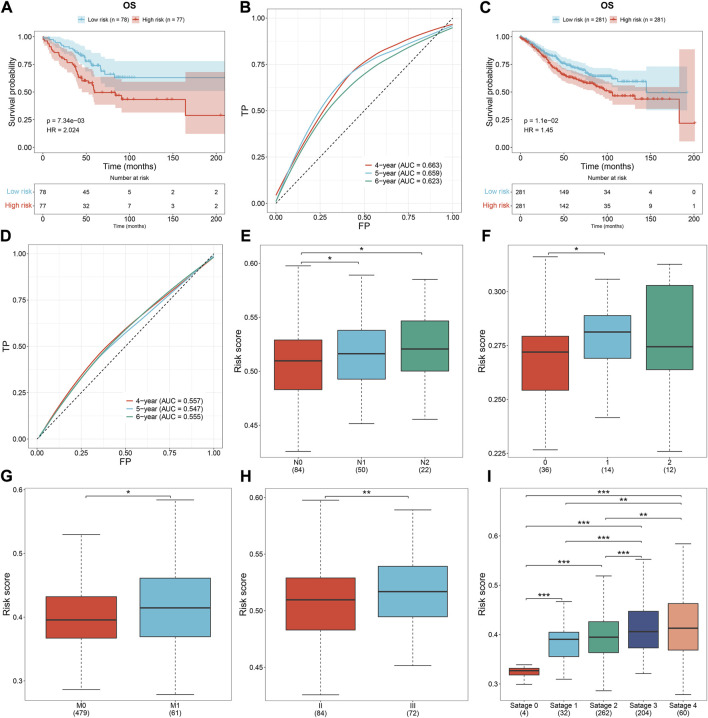
External verification of the oxidative stress-related gene signature. **(A,B)** Survival analysis and ROCs under 4-, 5-, and 6-year survival in the GSE103479 dataset. **(C,D)** Survival analysis and ROCs under 4-, 5-, and 6-year survival in the GSE39582 dataset. **(E,F)** Risk score across the N stage in the **(E)** GSE103479 and **(F)** GSE12945 datasets. **(G)** Risk score across the M stage from the GSE39582 dataset. **(H,I)** Risk score across the pathological stage in the GSE103479 and GSE39582 datasets. *p < 0.05; **p < 0.01; ***p < 0.001. GSE103479: *N* = 156; GSE39582: *N* = 562; GSE12945: *N* = 62.

To prove the robustness of the oxidative stress-related gene signature, this study included three independent cohorts. The same formula was used for computing the risk score. Both in the GSE103479 and GSE39582 cohorts, the high-risk subset exhibited worse OS than the low-risk subset, with relatively high AUCs at 4-, 5- and 6-year survival (Figures 5A–D). As the N stage (Figures 5E,F), M stage (Figure 5G), and pathological stage (Figures 5H,I) worsened, the risk score gradually increased. The aforementioned data demonstrated that the signature exhibited excellent robustness on distinct platforms.

### The oxidative stress-related gene signature correlates to antitumor immunity of CRC

**FIGURE 6:**
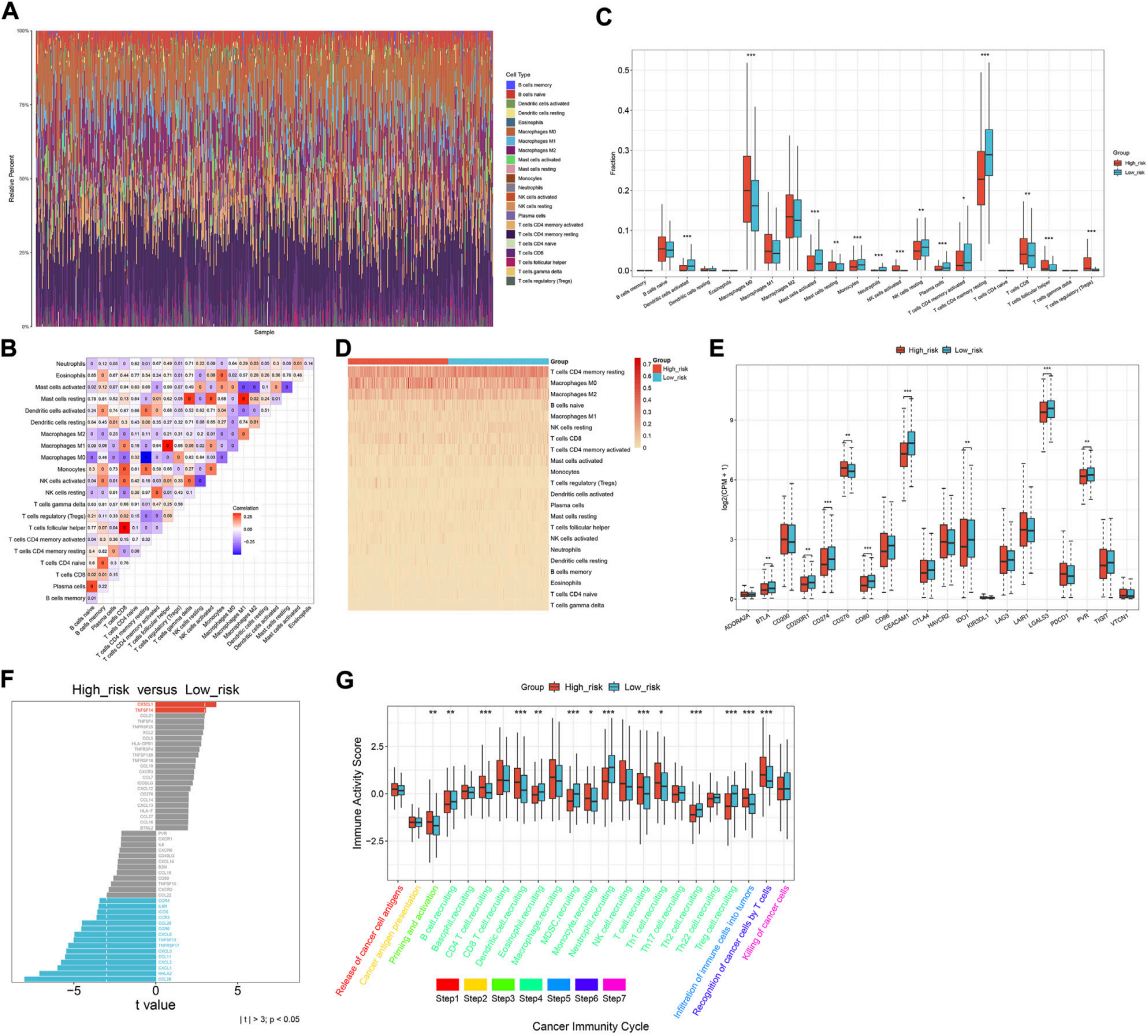
Oxidative stress-related gene signature correlates to the antitumor immunity of CRC. **(A)** Relative percent of the infiltration levels of immune cells across TCGA CRC. **(B)** Correlations between different immune cell populations. **(C,D)** Comparison of the infiltration of immune cells between subsets. **(E)** Comparison of immune checkpoints between subsets. **(F)** Difference in the expression of immunomodulators between subsets. **(G)** Difference in the activity of cancer immunity cycle between subsets. *p < 0.05; **p < 0.01; ***p < 0.001. *N* = 597.

Through the CIBERSORT approach, we estimated the immune cell infiltration across CRC specimens (Figures 6A,B). Activated dendritic cells, activated mast cells, monocytes, neutrophils, resting NK cells, plasma cells, activated memory T-cell CD4, and resting memory T-cell CD4 were significantly lower in the high-risk subset than those in the low-risk subset (Figures 6C,D). Meanwhile, M0 macrophages, activated NK cells, T-cell CD8, T-cell follicular helper, and T-cell regulatory (Tregs) exhibited elevated infiltration in the high-risk subset. The expression of immune checkpoints (*BTLA*, *CD274*, *CEACAM1*, *IDO1*, *LGALS3*, and *PVR*) exhibited down-regulation in the high-risk subset (Figure 6E). Additionally, immunomodulators (*IL6R*, *ICOS*, *CCR3*, *CCL20*, *CCR6*, *CXCL6*, *TNFSF13*, *TNFRSF17*, *CXCL3*, *CCL11*, *CXCL2*, *CXCL1*, *HHLA2*, and *CCL28*) were down-regulated in the high-risk subset (Figure 6F). Meanwhile, higher *CX3CL1* and *TNFSF14* expressions were found in the high-risk subset than in the low-risk subset. High activity of priming and activation, recruitment of CD4 T cells, dendritic cells, T cells, and Th1 cells; infiltration of immune cells into tumors; and recognition of cancer cells by T cells were found in high-risk subset compared to the low-risk subset (Figure 6G). In contrast, B-cell recruitment, eosinophil recruitment, MDSC recruitment, neutrophil recruitment, Th2 cell recruitment, and Treg cell recruitment showed lower activity in high-risk subset than the low-risk subset. Overall, the oxidative stress-related gene signature was correlated to antitumor immunity of CRC.

### Difference in CNV and mutation between high- and low-risk subsets

**FIGURE 7:**
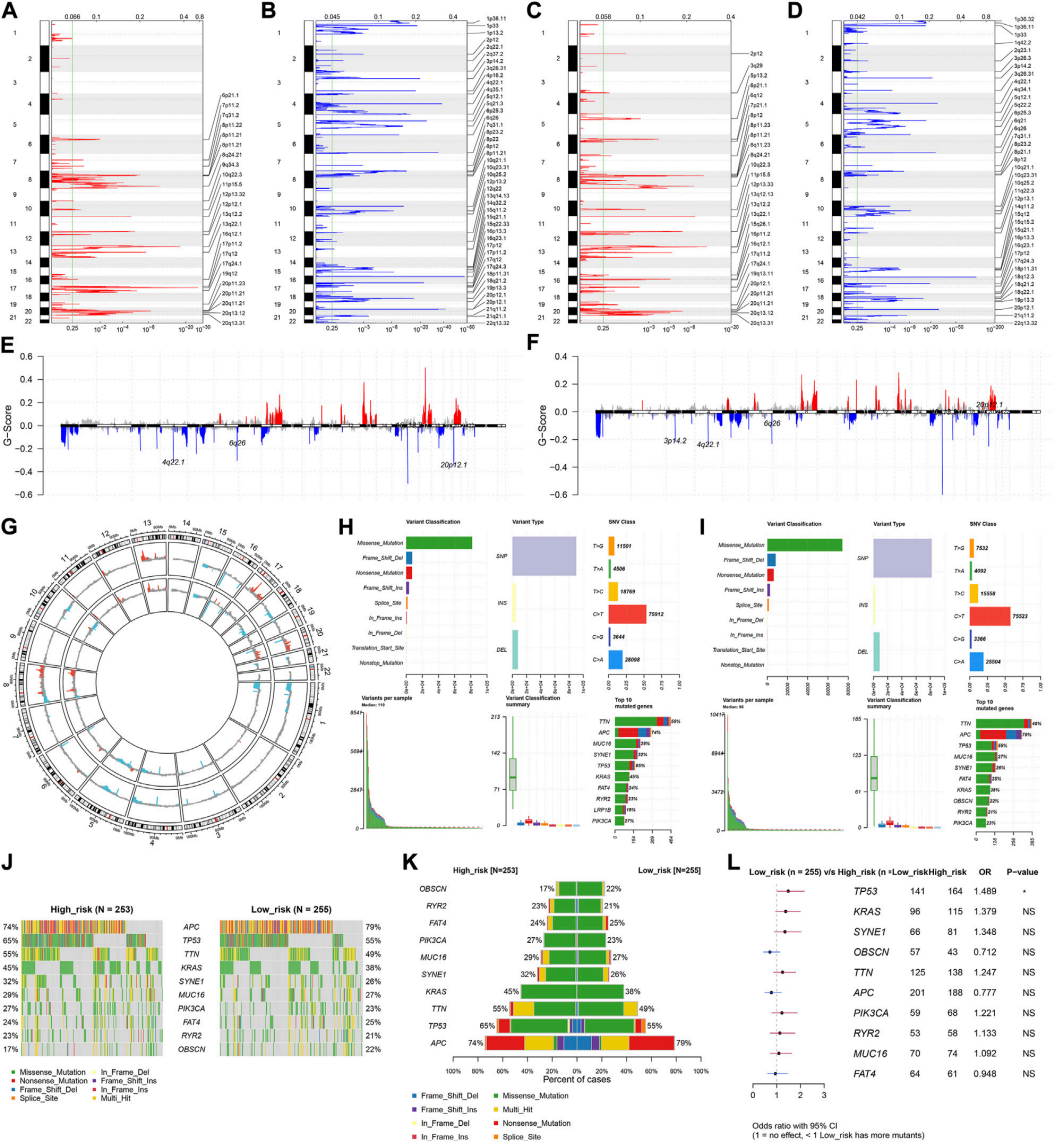
Differences in CNV and mutation between high- and low-risk subsets. **(A–D)** Copy number gains and deletions identified in **(A,B)** high- and **(C,D)** low-risk subsets by GISTIC 2.0. X-axis represents the CNV fractions on each chromosome, and y-axis represents the chromosome number. The mutation location on the chromosome is marked on the right side. Red and blue represent the significantly amplified and deleted regions, respectively. **(E–G)** Significant gains and deletions of copy number in high- and low-risk subsets. **(H,I)** Landscape of mutations in high- and low-risk subsets. **(J,K)** Difference in the frequencies of the top 10 mutated genes between subsets. **(L)** Forest plot of the correlation between mutated genes and high-risk. *N* = 597.

For the CNV data, we used GISTIC 2.0 to determine 24 amplified fragments and 44 deleted fragments in the high-risk subset (Figures 7A,B). Meanwhile, 28 amplified fragments and 40 deleted fragments were identified in the low-risk subset (Figures 7C–G). Compared with the high-risk subset, higher mutated frequencies of *APC*, *FAT4*, and *OBSCN* occurred in the low-risk subset (Figures 7H–K). In contrast, *TP53*, *TTN*, *KRAS*, *SYNE1*, *MUC16*, *PIK3CA*, and *RYR2* had higher mutated frequencies in the high-risk subset. Additionally, mutated *TP53* was significantly correlated to high-risk CRC (Figure 7L).

### Difference in drug sensitivity between high- and low-risk subsets

**FIGURE 8:**
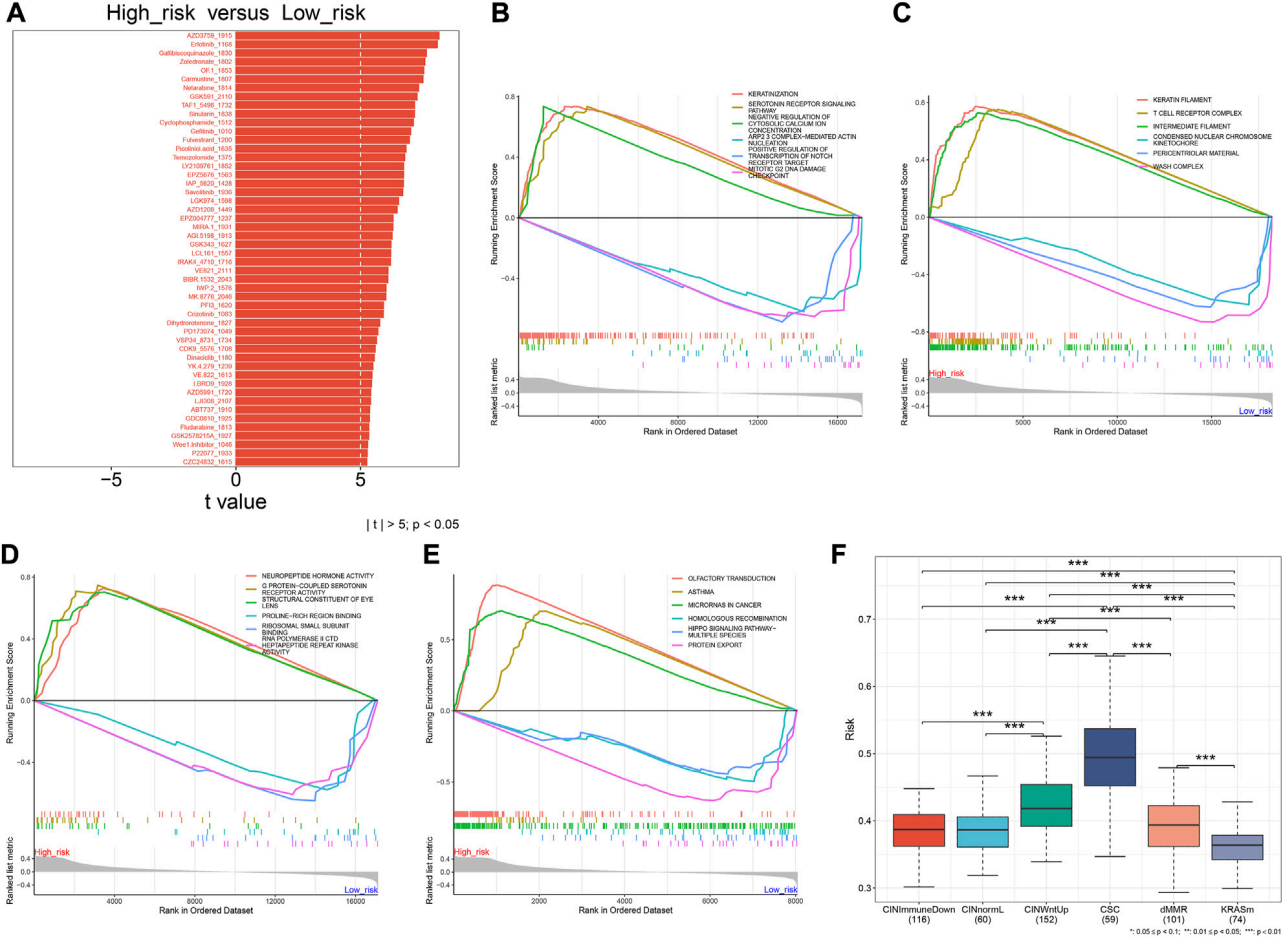
Oxidative stress-related gene signature is linked to drug sensitivity, signaling pathways, and molecular subtypes of CRC. **(A)** Difference in sensitivity to the top 50 drugs between high- and low-risk subsets. **(B–E)** Difference in biological processes, cellular component, molecular function, and KEGG pathways between subsets. **(F)** Difference in the risk score among molecular subtypes. ***p < 0.001. *N* = 597.

Drug sensitivity was analyzed between high- and low-risk subsets. The top 50 drugs were as follows: AZD3759, erlotinib, gallibiscoquinazole, zoledronate, OF.1, carmustine, nelarabine, GSK591, sinularin, TAF1, cyclophosphamide, gefitinib, fulvestrant, picolinici acid, temozolomide, IAP, LY2109761, EPZ5676, savolitinib, LGK974, AZD1208, MIRA.1, EPZ004777, AGI.5198, GSK343, LCL161, IRAK4, BIBR.1532, VE821, IWP.2, MK.8776, PFI3, crizotinib, dihydrorotenone, PD173074, VSP34, CDK9, dinaciclib, YK.4.279, VE.822, I.BRD9, LJI308, AZD5991, ABT737, GDC0810, fludarabine, GSK2578215A, Wee1.Inhibitor, P22077, and CZC24832 (Figure 8A).

### Difference in signaling pathways and molecular subtypes between high- and low-risk subsets

Molecular mechanisms involved in the oxidative stress-related gene signature were further explored. The risk score was significantly correlated with biological processes (keratinization, serotonin receptor signaling pathway, cytosolic calcium ion concentration, ARP 2/3 complex-mediated actin nucleation, positive regulation of transcription of the notch receptor target, and mitotic G2 DNA damage checkpoint; Figure 8B); cellular components of keratin filament, T-cell receptor complex, intermediate filament, condensed nuclear chromosome kinetochore, pericentriolar material, and WASH complex (Figure 8C); molecular functions of neuropeptide hormone activity, G protein-coupled serotonin receptor activity, structural constituent of eye lens, proline-rich region binding, ribosomal small subunit binding, and RNA polymerase II CTD heptapeptide repeat kinase activity (Figure 8D); KEGG pathways of olfactory transduction, asthma, microRNAs in cancer, homologous recombination, Hippo signaling pathway-multiple species, and protein export (Figure 8E). Additionally, there was a remarkable difference in the risk score among different molecular subtypes (CIN, CSC, dMMR, and KRASm) of CRC (Figure 8F). Particularly, the CSC subtype had the highest risk score.

### Experimental verification of the genes within the oxidative stress-relevant gene signature

**FIGURE 9:**
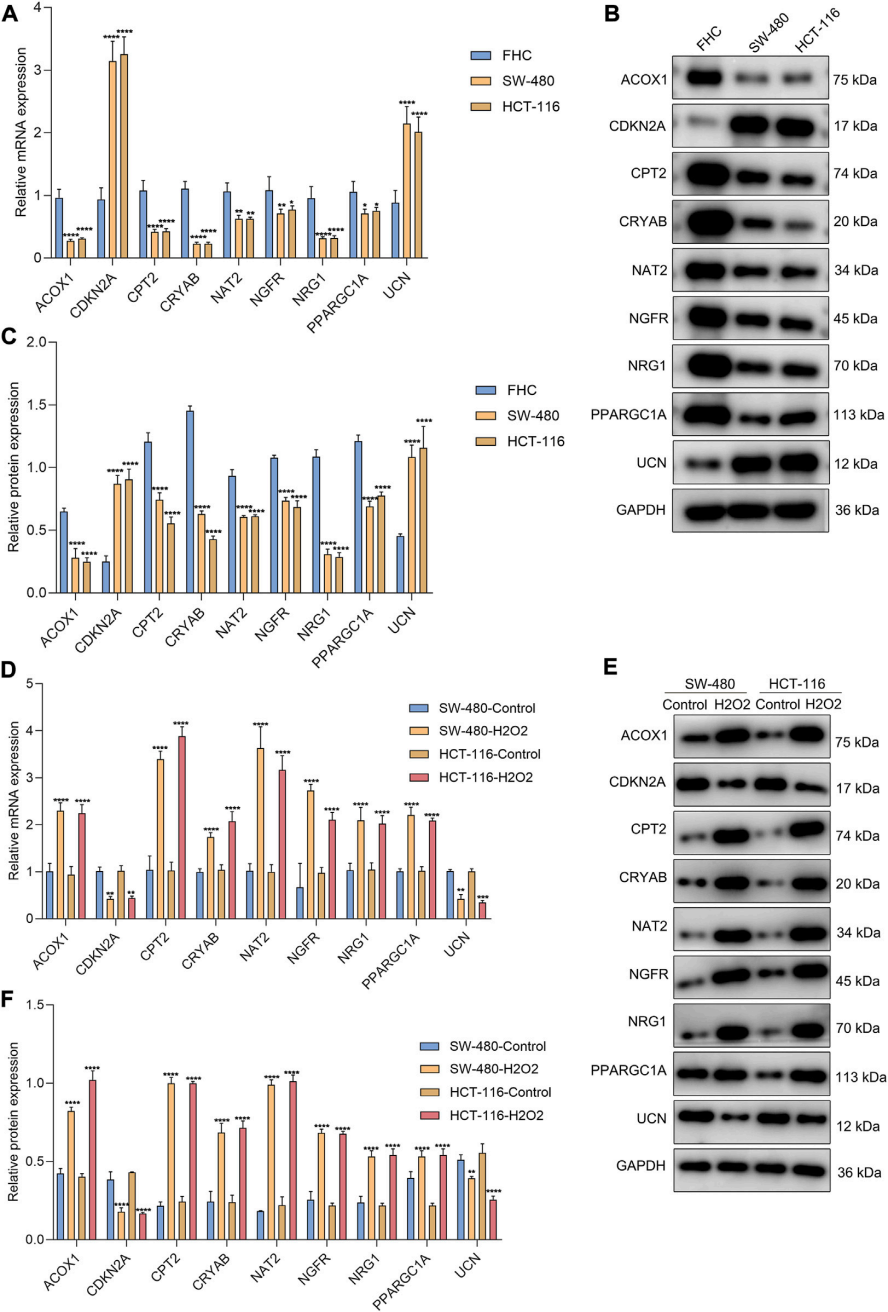
Experimental verification of the genes from the oxidative stress-relevant gene signature. **(A–C)** RT-qPCR and Western blot for the expression of *ACOX1*, *CPT2*, *NAT2*, *NRG1*, *PPARGC1A*, *CDKN2A*, *CRYAB*, *NGFR*, and *UCN* in FHC, SW-480, and HCT-116 cells. **(D–F)** RT-qPCR and Western blot for their expression in H_2_O_2_-induced SW-480 and HCT-116 cells. *p < 0.05; **p < 0.01; ***p < 0.001; ****p < 0.0001. *N* = 3.

The genes in the signature were verified in the human colorectal mucosal cell line (FHC) along with CRC cell lines (SW-480 and HCT-116) through RT-qPCR or Western blot. *CDKN2A* and *UCN* were up-regulated and *ACOX1*, *CPT2*, *NAT2*, *NRG1*, *PPARGC1A*, *CRYAB*, and *NGFR* were down-regulated in CRC than normal cells (Figures 9A–C). Next, we further validated the relationships between the genes in the signature and oxidative stress. After exposure to H_2_O_2_, their expression was measured in SW-480 and HCT-116 cells. Higher expression of *ACOX1*, *CPT2*, *NAT2*, *NRG1*, *PPARGC1A*, *CRYAB*, and *NGFR* as well as lower expression of *CDKN2A* and *UCN* were observed in H_2_O_2_-induced CRC cells (Figures 9D–F), indicating the relevance of oxidative stress during CRC development.

## Discussion

Oxidative stress-related signatures have been established in acute myeloid leukemia (Dong et al., 2021), melanoma (Yang et al., 2021), clear-cell renal cell carcinoma (Wu Y. et al., 2021a), gastric cancer (Wu Z. et al., 2021b), head and neck squamous cell carcinoma (Wang and Zhou, 2021), glioma (Lu et al., 2021), and bladder cancer (Zhang et al., 2022). Alterations in redox status accompanied by increased production of ROS have been implicated in CRC (Lee et al., 2021). Nevertheless, so far, no oxidative stress-related model has been proposed for CRC. Considering the fact that oxidative stress is a complex process involving different genes, in the present study, we proposed an oxidative stress-related gene signature composed of *ACOX1*, *CPT2*, *NAT2*, *NRG1*, *PPARGC1A*, *CDKN2A*, *CRYAB*, *NGFR*, and *UCN* to predict CRC patients’ clinical outcomes with the LASSO approach.

Reliable markers in predicting immunotherapeutic responses of CRC patients are still insufficient in clinical practice (Chen L. et al., 2021b). Dual suppression of endoplasmic reticulum stress and oxidation stress may manipulate macrophage polarization following hypoxia to enhance immunotherapeutic sensitivity (Jiang et al., 2021). *SENP7* can sense oxidative stress to maintain metabolic fitness and antitumor effects of CD8^+^ T cells (Wu et al., 2022). Moreover, altered tumor metabolism *via* CD4^+^ T cells results in TNF-α-dependent intensified oxidative stress and tumor cell deaths (Habtetsion et al., 2018). The oxidative stress-related gene signature was correlated with the antitumor immunity of CRC, indicating that this signature might enable prediction of the immunotherapeutic response (Liu et al., 2020). Genomic alterations and CNVs were compared between high- and low-risk subsets. Particularly, the high-risk subset was remarkably linked to more aggressive molecular alteration: mutated *TP53* that triggers enhanced proliferative capacity *via* consuming oxygen and producing abnormal vasculature during the early stage of cancer development. There were remarkable differences in drug sensitivity between high- and low-risk subsets. Additionally, the risk score was linked to CRC-related pathways, such as mitotic G2 DNA damage checkpoint, microRNAs in cancer, and Hippo signaling pathway.

Our experimental studies demonstrated that *CDKN2A* and *UCN* were up-regulated and *ACOX1*, *CPT2*, *NAT2*, *NRG1*, *PPARGC1A*, *CRYAB*, and *NGFR* were down-regulated in CRC cells (SW-480 and HCT-116) compared with human colorectal mucosal cells (FHC). In H_2_O_2_-induced CRC cells, their expression was remarkably altered. Butyrate-induced colonocyte differentiation determines *CDKN2A* as a prognostic biomarker of CRC recurrence (Dasgupta et al., 2019). Patients who have tumor chromosomal *CDKN2A* deletion are prone to immunotherapeutic resistance (Horn et al., 2018). *ACOX1* may attenuate the migration and invasion of CRC cells (Sun et al., 2017). Down-regulated *CPT2* induces stemness and oxaliplatin resistance in CRC through the ROS/Wnt/β-catenin-triggered glycolytic metabolism (Li et al., 2021). Additionally, its down-regulation heightens proliferation and weakens apoptosis *via* p53 signaling in CRC (Liu et al., 2022). *NAT2* down-regulation is also found in CRC, which correlates to CRC patients’ metastasis and survival (Zhu et al., 2021). *CRYAB* correlates to clinical outcomes and immunocyte infiltrations in CRC (Deng et al., 2021). *NGFR* improves the chemosensitivity of CRC cells by strengthening the apoptotic and autophagic effects of 5-fluorouracil by activating *S100A9* (Chen H. et al., 2021a). Combining previous evidence, the genes in the oxidative stress-related signature play essential roles in CRC progression.

Our analysis is a retrospective study, resulting in unavoidable limitations. As many datasets as possible were included, so sampling bias from tumor heterogeneity and different platforms can only be decreased, but not completely removed. Although we experimentally validated the genes from the oxidative stress-relevant gene signature, more experimental studies are needed for clarifying the functional significance of oxidative stress in CRC.

## Conclusion

In summary, this study proposed an oxidative stress-related signature composed of *ACOX1*, *CPT2*, *NAT2*, *NRG1*, *PPARGC1A*, *CDKN2A*, *CRYAB*, *NGFR*, and *UCN* to predict clinical outcomes and therapeutic responses of CRC patients, which provided valuable information for understanding the functional roles of oxidative stress in CRC development, assisting prognosis prediction and guiding adjuvant therapy (especially small molecular compounds and immunotherapy), thereby facilitating precision oncology of CRC.

## Data Availability

The datasets presented in this study can be found in online repositories. The names of the repository/repositories and accession number(s) can be found in the article/Supplementary Material.

## Abbreviations

CRC, colorectal cancer; TCGA, The Cancer Genome Atlas; GEO, Gene Expression Omnibus; OS, overall survival; DFS, disease-free survival; DSS, disease-specific survival; PFS, progression-free survival; ROS, reactive oxygen species; CRC, colorectal cancer; COAD, colon adenocarcinoma; READ, rectum adenocarcinoma; CNV, copy number variation; LASSO, least absolute shrinkage and selection operator; ROCs, receiver operator characteristic curves; AUC, area under the curve; CIBERSORT, Cell Type Identification by Estimating Relative Subsets of RNA Transcripts; GDSC, Genomics of Drug Sensitivity in Cancer; GSEA, Gene set enrichment analysis; GO, Gene Ontology; KEGG, Kyoto Encyclopedia of Genes and Genomes; cDNA, complementary DNA.
