# Supplementary figures and images for: Establishment and external verification of an oxidative stress-related gene signature to predict clinical outcomes and therapeutic responses of colorectal cancer
Source: Front Pharmacol. 2023 Feb 13;13:991881. doi: 10.3389/fphar.2022.991881 (PMC9968941; doi:10.3389/fphar.2022.991881)

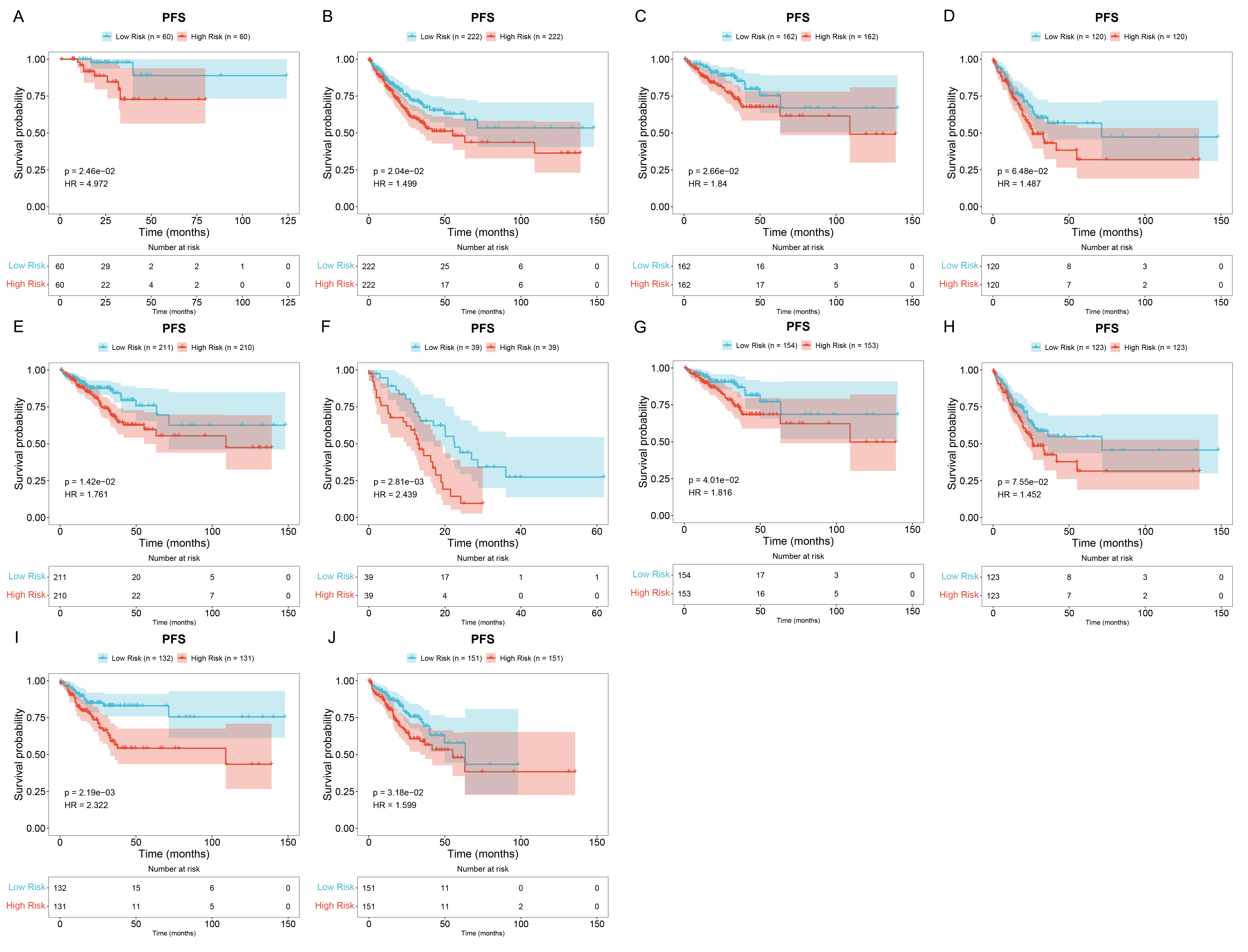

Supplement: Supplementary file 3 [file Image3.TIF]

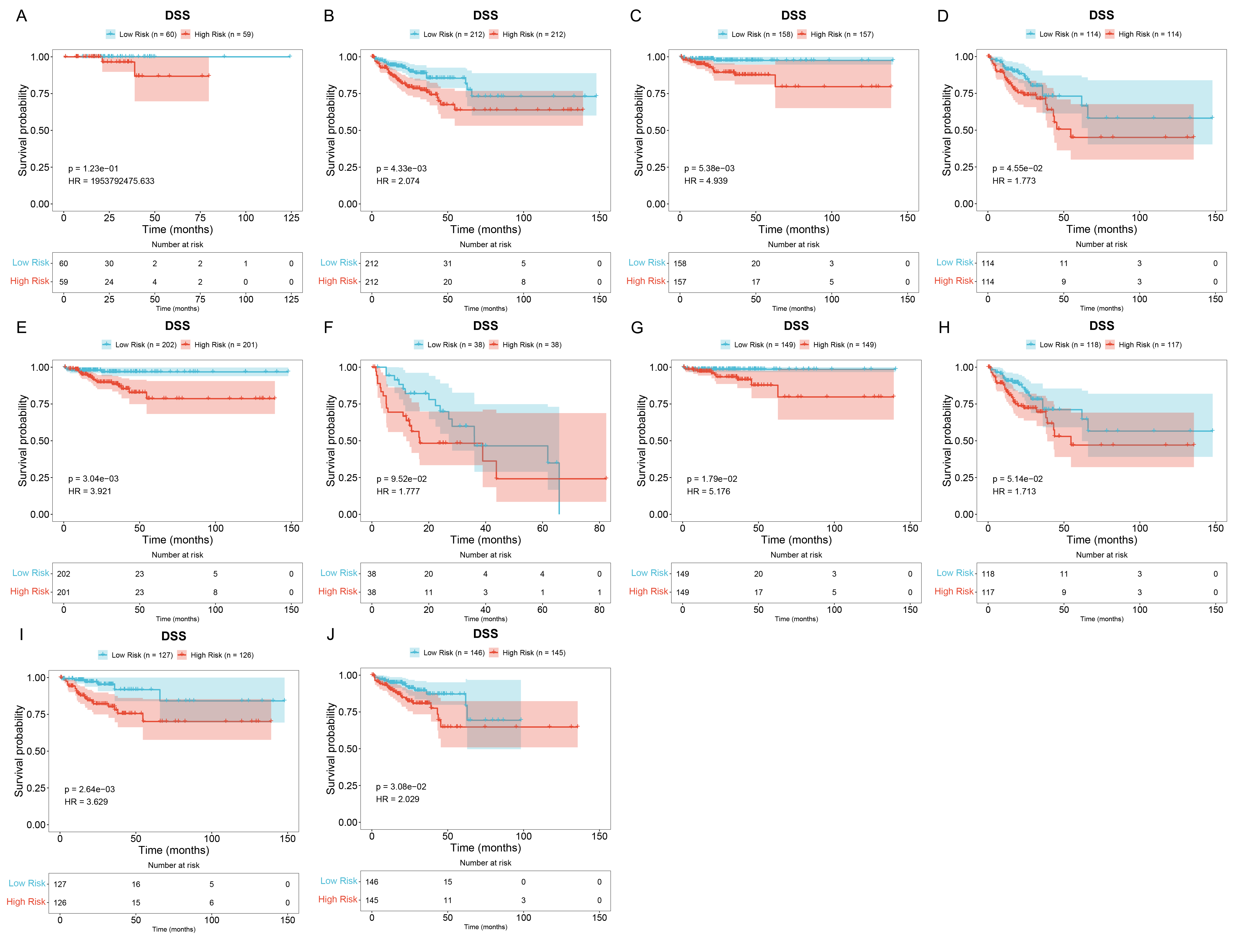

Supplement: Supplementary file 4 [file Image2.TIF]

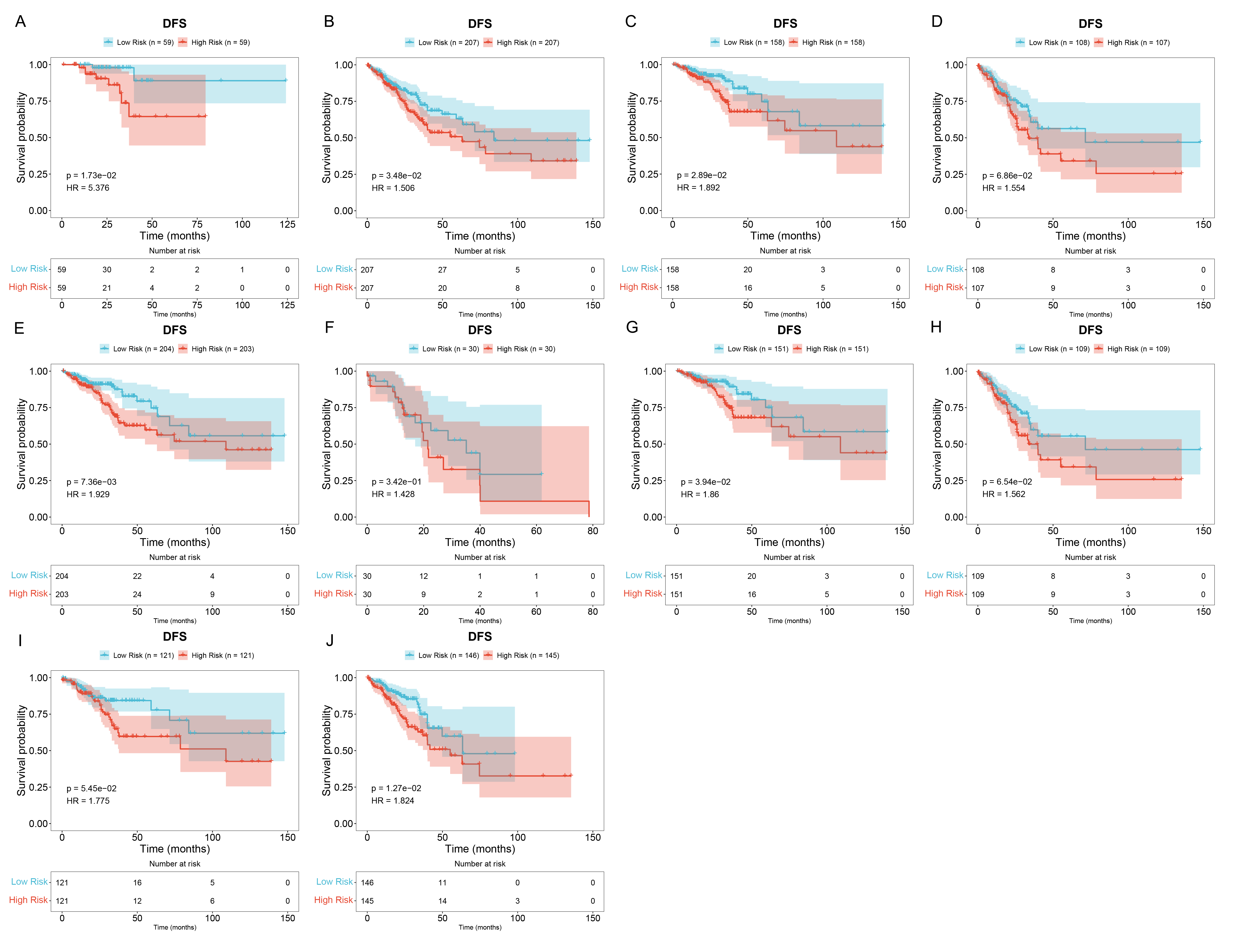

Supplement: Supplementary file 5 [file Image1.TIF]
